# Development of an AI-based image/ultrasonic convergence camera system for accurate gas leak detection in petrochemical plants

**DOI:** 10.1016/j.heliyon.2024.e28905

**Published:** 2024-03-30

**Authors:** JoonHyuk Lee, YoungSik Kim, Abdur Rehman, InKwon Kim, JaeJoon Lee, HongSik Yun

**Affiliations:** a Korean Fire Protection Association, Seoul, 07328, South Korea; b Interdisciplinary Program in Crisis, Disaster and Risk Management, Sungkyunkwan University, 2066, Seobu-ro, Jangan-gu, Suwon-si, Gyeonggi-do, 16419, South Korea; c Stratio, Inc., Seongnam-si, Gyeonggi-do, 13449, South Korea; d Sound Camera Business/Software Lab., SM Instruments, Inc., Daejeon, 34109, South Korea; e Department of Fire safety Engineering, Jeonju University, 303, Cheonjam-ro, Wansan-gu, Jeonju-si, Jeollabuk-do, 55069, South Korea

**Keywords:** Pipe leak, Petrochemical plant, Ultrasonic, Deep learning, Object detection

## Abstract

Outdoor pipeline leaks are difficult to accurately measure using existing concentration measurement systems installed in petrochemical plants owing to external air currents. Besides, leak detection is only possible for a specific gas. The purpose of this study was to develop an image/ultrasonic convergence camera system that incorporates artificial intelligence (AI) to improve pipe leak detection and establish a real-time monitoring system. Our system includes an advanced ultrasonic camera coupled with a deep learning-based object-detection algorithm trained on pipe image data from petrochemical plants. The collected data improved the accuracy of detected gas leak localization through deep learning. Our detection model achieves an mAP_50_ (Mean average precision calculated at an intersection over union (IoU) threshold of 0.50)score of 0.45 on our data and is able to detect the majority of leak points within a system. The petrochemical plant environment was simulated by visiting petrochemical plants and reviewing drawings, and an outdoor experimental demonstration site was established. Scenarios such as flange connection failure were set under medium-/low-pressure conditions, and the developed product was experimented under gas leak conditions that simulated leakage accidents. These experiments enabled the removal of potentially confounding surrounding noise sources, which led to the false detection of actual gas leaks using the AI piping detection technique.

## 1 Introduction

The increase in chemical leakage accidents, especially in petrochemical plants, has led to concerns about human casualties reaching 30%. After safety management was relaxed, a noticeable increase in the number of chemical accidents revealed that more effective prevention and detection systems were required. The number of chemical accidents in Korea increased from 58 in 2019 to 75 in 2020 and 93 in 2021 [1]. Flammable gas leaks can lead to major accidents, including fires and explosions, which can severely damage property [2,3].

Most leaks occurred at the joints and frequently involved the flanges, valves, and connectors among the equipment components of the Korean petrochemical plant that was investigated. Flange leaks occurred most frequently [4].

Therefore, to detect leaks occurring at flanges and joints, research focusing on leaks at joint points in important infrastructures through which fluids such as water are transported outside of the petrochemical industry has been conducted. Ayed et al. [5] conducted a leak location analysis that analyzed temporal changes in pressure signals and Brunone et al. [6] presented new methods for performance evaluation and design through numerical analysis. As a result, we can clearly see that the leak detection technology needs to be improved.

In this study, flange leakage was selected as the specific research scenario. To prevent leaks occurring in joint components, such as flanges, various detection systems for application in petrochemical plants are being researched and developed [[7], [8], [9], [10], [11]]. However, detection technologies remain inadequate.

Petrochemical plants are located outdoors, and leaked gas can be dispersed by air currents; therefore, it is difficult to quickly and precisely detect gas leaks occurring in low- and medium-pressure gas pipes using existing systems and on-site inspections. The detection of leaks is also complicated in petrochemical plants that handle multiple gas types because some types of gas cannot be detected by sensors, including sensors conventionally used to measure gas concentrations.

Currently, gas detection units have been installed in petrochemical plants to measure gas concentrations in accordance with national standards. In Korea, according to the “Technical Guidelines for Gas Leak Detector Installation and Maintenance” (Kosha Guide P-166-2020), one or more gas-leak detectors are installed in adjacent locations with a high risk of leakage. The placement standards were set according to the characteristics of each location [12]. However, in the case of gas leak detection alarms, the type of gas is regulated, and most methods incorporate concentration thresholds. Because the gas leak detectors for petrochemical plants are installed outdoors, their detection efficacy is low owing to air currents and a lack of barriers to diffusion.

Alternatives, such as ultrasonic detection cameras and thermal imaging cameras (Optical Gas Imaging, OGIs), have been used in this field. Although they are portable and can be used by a person to record measurements [13], their inherent disadvantage is that 24-h monitoring is not possible. In addition, the operator's judgment of the detection results is required [14].

Ultrasonic measurements can be used outdoors, where wind and diffusion rapidly disperse leaking gas. Gas sensors, which are commonly used in fields that operate on the basis of chemical reactions, are difficult to operate in these environments. Infrared methods, including OGIs, which are widely used in the field, respond only to specific gases. The technology used in this study leverages the vortex formation that occurs when gas leaks from a pipe and captures an ultrasonic band signal through the microphone sensor built into the camera. Thus, gas leaks can be detected regardless of gas type [15]. Thus, we combined a deep-learning-based object detection algorithm with an ultrasound camera and installed this equipment at our experiment site. This study represents an innovative attempt to build a surveillance monitoring system through the convergent use of these technologies.

Through the system proposed in this study, it was possible to secure advanced performance such as increased precision through experiments on various pipe leak situations using artificial intelligence (AI) learning based on ultrasound/image data collection. The focus of this research was to develop a technology that detects leaks and their locations by training artificial intelligence using the obtained image data. The developed technology can provide a safer environment, in conjunction with a safe system for petrochemical plants.

## 2 Literature review

The petrochemical industry is one of the most dangerous industries because it involves a wide variety of processes for the transport, storage, and use of hazardous materials [16,17]. The potential safety hazards in the petrochemical industry include fires, explosions, and toxic emissions [[18], [19], [20], [21], [22]]. Many gas leaks occur because of faulty pipelines, which have resulted in significant economic losses and environmental damage [[23], [24], [25], [26], [27]]. Several researchers have proposed techniques for detecting and mitigating pipeline leakages [[28], [29], [30], [31]].

Technologies for detecting gas leaks continue to progress. Technologies for detecting gas leaks using ultrasonic waves have been developed recently [[32], [33], [34], [35], [36]]. These technologies are based on the principle that ultrasonic waves are generated by turbulent flow when a gas leak occurs in a pipeline [37]. Farooqui et al. [38] demonstrated that an ultrasonication-based well-leak detector could efficiently detect small tube and casing leaks. Furthermore, steam leakage ejected from a nozzle at constant pressure was identified using a microphone sensor [[39], [40], [41]]. A study [42] was also conducted to validate an ultrasonic gas-leak localization method based on a sensor array.

Cao et al. [43] found that the gas leakage signal is significantly different from the baseline signal in an ultrasonic frequency band. They found that the leakage noise was concentrated at lower frequencies and could be transmitted through pipelines [44].

A technique for detecting leaks by attaching ultrasonic acoustic sensors to pipes was proposed [45,46]. Seo and Lee [47] proposed the post-processing of ultrasonic reception signals to detect leaks in gas fuel tanks, which depended on other sensors, such as pressure sensors. Scheuer and DeCorby [48] performed ultrasonic emission in the MHz range using nitrogen gas and concluded that gas leaks could be sensed both off-axis and off-position. Although ultrasonic detection technology holds promise, certain implementation limitations exist, including the inability to cover large areas when sensors are attached directly to pipes. Cho et al. [49] fabricated and tested a remote-measurable ultrasonic gas leak detection unit and suggested the use of deep learning as a method for future development.

Recently, deep learning has achieved tremendous success, enabling breakthroughs in domains such as speech, text, and computer vision. Deep learning development began in 2012, when the convolutional neural network (CNN)- [50] based architecture AlexNet [51] outperformed its competitors in the ImageNet classification challenge [52]. AlexNet has ignited the widespread adoption of deep learning algorithms. The ability of deep-learning models to learn meaningful representations from raw data, coupled with their scalability for computational resources, has paved the way for numerous applications.

Object detection is a fundamental task in computer vision that involves the classification and localization of an object within an image. R–CNN [53] was the first study to show that a CNN can dramatically improve object detection compared with traditional algorithms. Fast R-CNNs [54] and Faster R-CNNs [55] were later proposed, offering improved accuracy and speed for object detection. However, these improvements have been insufficient for practical real-time applications. However, with the introduction of you only look once (YOLO) [56] and single-shot detectors (SSD) [57], we have seen a paradigm shift in object detection. YOLO, owing to its real-time performance and accuracy, has attracted the attention of the research community and has become the cornerstone of object detection models.

The basic concept of YOLO is to divide an image within a grid and directly predict the bounding box and class probability from the grid. Researchers have built on this idea and proposed several improvements to the original architectures, including YOLO9000 [58], YOLOv3 [59], and YOLOv4 [60]. Key improvements to the original architecture include the adoption of anchor boxes for handling objects of various sizes and the inclusion of a feature pyramid network (FPN) [61], path aggregation network (PAN) [62], and spatial attention module (SAM) to enhance object detection at different scales and feature representations. The authors also improved the backbone architecture.

Beyond academia, deep learning-based algorithms have found extensive applications across various industries. One of these crucial applications is the detection of gas leaks, which pose a threat to human life, environment, and other valuable assets. Researchers have recognized the potential of deep learning for accurate and early gas leak detection, enabling proactive measures to prevent unfortunate events caused by such leaks [[63], [64], [65], [66], [67]]. Wang et al. [68] used optical gas imaging (OGI) along with a convolutional neural network (CNN) to detect gas leakage from an image. Similarly, Melo et al. [69] proposed the use of normal closed-circuit camera (CCTV) videos for gas-leak classification. These CNN-based approaches only classify gas leaks. However, they could not localize the leak. With improvements in real-time object detection algorithms, researchers have rapidly utilized them for this task. Shi et al. [70] used an OGI with Faster R–CNN [55] for pipe leak detection. In another study [71], researchers compared the efficacy of YOLOv4 [60] and Faster R–CNN [55] for pipe leak detection. Ahmad et al. [72] conducted a study based on acoustic imaging and deep learning and obtained a higher accuracy than that seen in previous studies. However, the proposed method can only detect leaks, and cannot provide information regarding leak localization.

The state of research has advanced to a stage where we can attempt to detect a leak source, such as a joint, from a distance, by merging ultrasonic gas leak detection technology with artificial intelligence (AI) object detection technology to overcome ambient noise [73]. However, to-date, there has been a lack of empirical research verifying the performance of developed products. In addition, when ultrasonic gas leak detection technology and AI object detection are combined, there is a lack of diversity in the pipe leak points that are subject to object detection. In this study, we investigated the points at which leaks were expected, divided these areas into more detailed classes, and performed object detection.

In this paper, we propose a sensor that combines these two modalities. Ultrasonic sound waves were detected using an ultrasonic camera. Subsequently, we applied the YOLO object detector to a normal camera feed captured by the same ultrasonic camera. Our goal was to align the spatial outputs of the two sensors to eliminate the noise or false alarms generated by the sound sensor. Through demonstration experiments, we confirmed that the developed system effectively removed noise and identified gas-leak sources.

## 3 System configuration

**Fig. 1:**
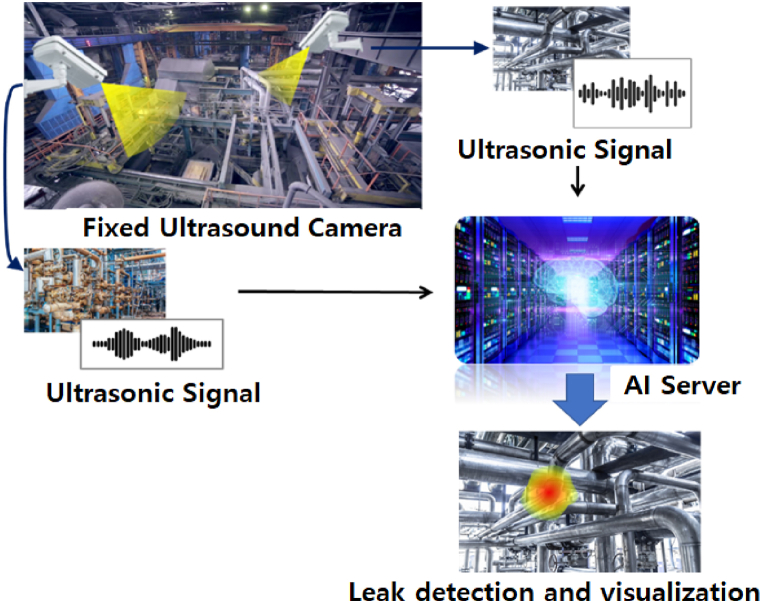
Schematic of our image/sound based AI convergence gas leak detection system configuration.

In this paper, we propose a microleak detection system for petrochemical plant piping using AI based on ultrasonic/image convergence hardware. This system installs ultrasonic and image-convergence sensors in the field, receives data, analyzes them using AI, visualizes the results, and transmits the detected images. As shown in Fig. 1, the main components of the system are an ultrasonic detection camera, AI server, and data storage server. In addition, it includes auxiliary facilities for monitoring and connecting networks.

The ultrasonic camera shown in Fig. 1, which is an element of the gas leak monitoring system, estimates the location of the leak using ultrasonic signals measured from multiple microphone sensors and visually marks the location in the image obtained with the optical camera. The hardware has a waterproof/dustproof function that accommodates the conditions of outdoor petrochemical plants.

**Fig. 2:**
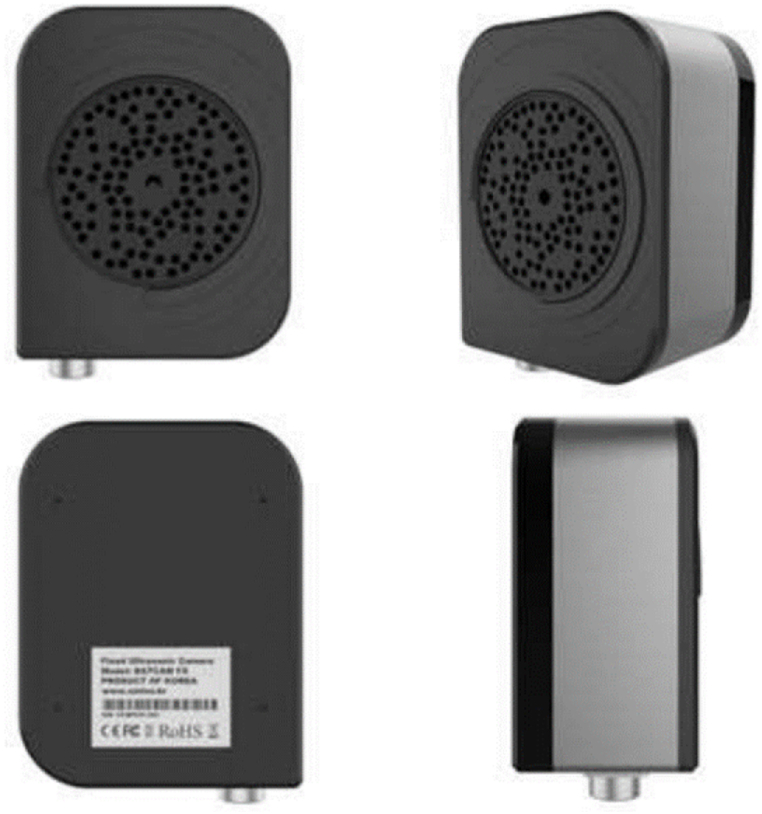
Design of the prototype.

The ultrasonic camera, which is an important element in gas leak monitoring systems, was designed in the power-over-ethernet (PoE) form to facilitate the outdoor connection of power and network lines for data communication. The camera was designed and manufactured as shown in Fig. 2.

## 4 Data for the object detection algorithm

**Fig. 3:**
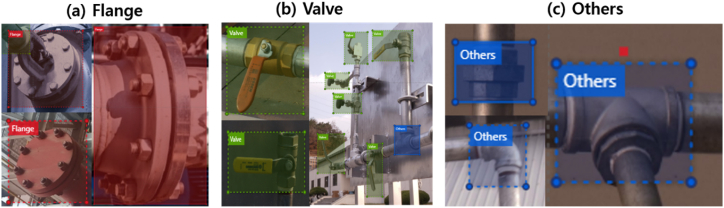
Illustrations of the different classes in our dataset.

Two sources were used for data collection. First, we manually collected images of different pipelines from the site where the gas-leak detection system was deployed. Simultaneously, we used a web crawling tool to collect industrial pipeline images from the web. Images with sizes of >300 × 300 pixels were selected. After data collection, the images were carefully labeled. After a careful examination of the collected data, we categorized our training data into three classes for object detection. We focused on detecting every joint within a pipeline image because joints are the most common points of failure in leaking gas systems. The three classes of joints identified in our data were: 1) flanges, 2) valves, and 3) others (Fig. 3). Flanges and valves were segregated into distinct classes, as shown in Fig. 3(a) and (b) respectively, owing to their ubiquitous presence and standardized configurations within industrial contexts. On the other hand, joint types beyond flanges and valves were labeled into a singular category denoted as “others,” as depicted in Fig. 3(c). In the “others” class, we encompassed all joint varieties employed in pipelines except for flanges or valves. Given the diverse range of these joints, we opted to amalgamate them into a unified category to streamline classification.

**Fig. 4:**
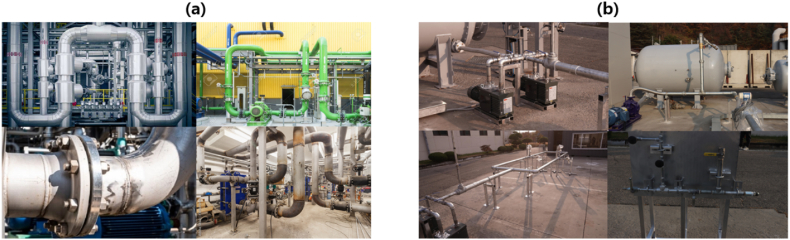
Data collection from two sources. (a) Data collected form web crawling; (b) Data collected from the experiment site.

As we collected data from two different sources (Fig. 4), we attempted to label objects that were clearly visible and of an appropriate size. Data from these two sources provide diverse information and helped us to generalize our detection model. Fig. 4 shows the dataset collected from the web(Fig. 4(a)) and the experiment site(Fig. 4(b)) where the solution was deployed. We collected 496 images; the numbers of instances per class are listed in Table 1.

**Table 1:** Instances of each class within our datasets.

Class	Instances
Valve	933
Flange	1422
Others	768

## 5 Object detection

Below, we discuss the details of our object detection algorithm architecture, implementation details, and study results.

### 5.1 Architecture

In our experiment, we used YOLOv5 [74] for object detection. YOLOv5 is based on CSPDarknet53 and similar to YOLOv4 [60]. CSPDarknet is a combination of cross-stage partial connections (CSP) and Darknet forms. The CSP [75] were designed to mitigate duplicate gradient information within a network during optimization. The input tensor was divided into two parts. One part was convolved once, and multiple convolution residual operations were applied in the second part.

**Fig. 5:**
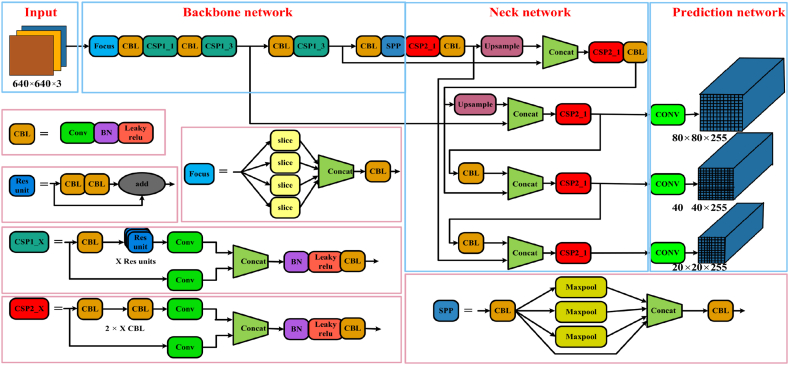
Basic architecture of YOLOv5 [74] as illustrated in Ref. [76].

To capture multiscale features, YOLOv5 integrates a neck module called the path aggregation network (PANet) [62]. PANet enhances feature representation by aggregating information from different stages of the backbone network. It utilizes lateral connections and upsampling to fuse low- and high-resolution feature maps, thereby enabling effective object detection across varying scales. The YOLOv5 architecture illustrated in Fig. 5. It consists of four parts: the input, backbone, neck, and prediction network/head.

The detection head in YOLOv5 is responsible for predicting bounding boxes and class probabilities. It consists of multiple detection layers that operate on different scales of feature maps. Each detection layer predicts a fixed number of bounding boxes, anchor box offsets, and associated class probabilities. These layers employ convolutional and activation operations to extract the relevant features and perform object detection.

### 5.2 Implementation details

We used YOLOv5 [74] weights pre-trained on COCO [77] as the starting point for training our dataset. We used an input image size of 1280 and trained for 150 epochs with a batch size of 16. A stochastic gradient descent (SGD) with a learning rate of 0.01 and momentum of 0.9 was used. Cosine learning rate decay was used during training. All other hyperparameters were the same as those used in the original implementation of YOLOv5. We used the YOLOv5 (L) model for all experiments and an 80/20 training/validation split during training.

### 5.3 YOLOv5 results

**Fig. 6:**
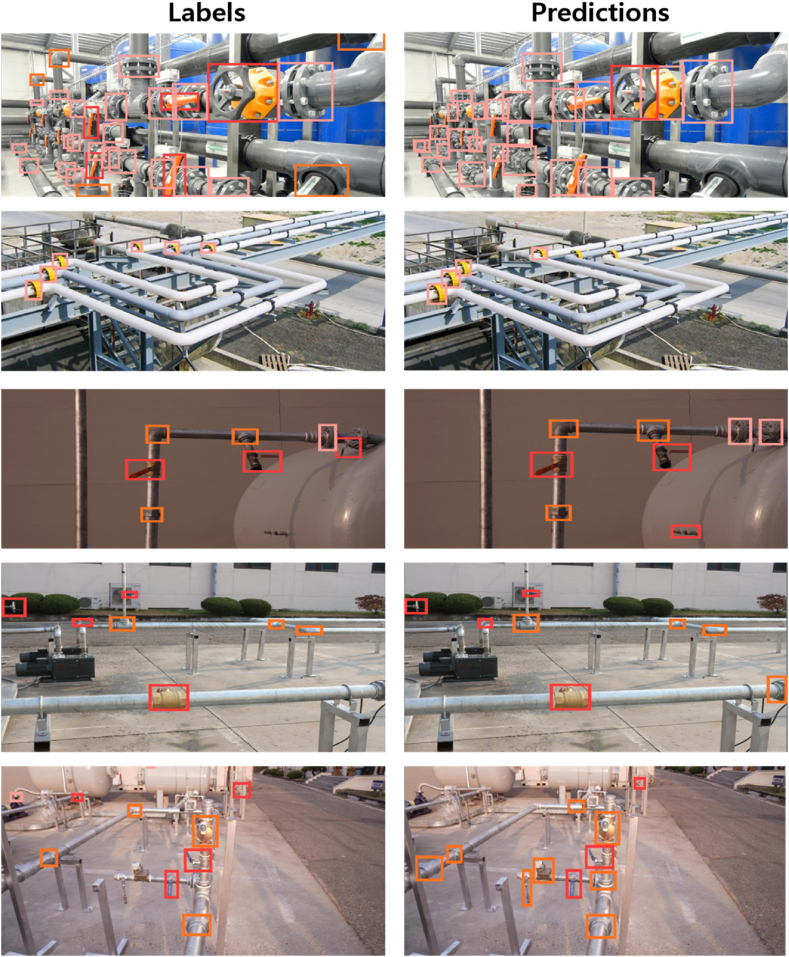
Labels and corresponding predictions for our detection model.

The object detection results were evaluated using precision, recall, F1 score, and mAP_50_ as parameters. The object detection results are listed in Table 2. The “Flange” class exhibited the highest mAP_50_ score (0.50), whereas the “Others” class exhibited the lowest score (0.411). Because the “Flange” class exhibited the least dataset variation, the flanges were easier to identify in an image. The “Others” class covered varying types and shapes of joints that do not have uniform distribution in our dataset. The overall mAP_50_ score of the proposed detection model across all the classes was 0.454. Achieving a high mAP_50_ score is challenging, because the dataset comprises objects with large variations in appearance, occlusion, and diverse backgrounds. Fig. 6 shows the labels and their corresponding predictions from the detection model.

**Table 2:** Summary of detection results achieved with the evaluation data.

Class	Precision	Recall	F1	mAP50
All	0.527	0.488	0.505	0.454
Valve	0.562	0.528	0.545	0.449
Flange	0.605	0.493	0.543	0.501
Others	0.415	0.442	0.428	0.411

The object detection model could detect and classify most joints in the evaluation images; however, some joints were misclassified as incorrect classes. This imperfect object detection model is still useful in our application because it is more important to detect possible leakage points than to accurately classify the exact type of joints. However, the object detection model can miss some joints, which can be critical in certain leakage situations. Additional techniques that can be applied in the system to reduce the chances of missing leaking points will be discussed later in Chapter 8.

## 6 Experimental set-up

### 6.1 Demonstration site design and construction

**Fig. 7:**
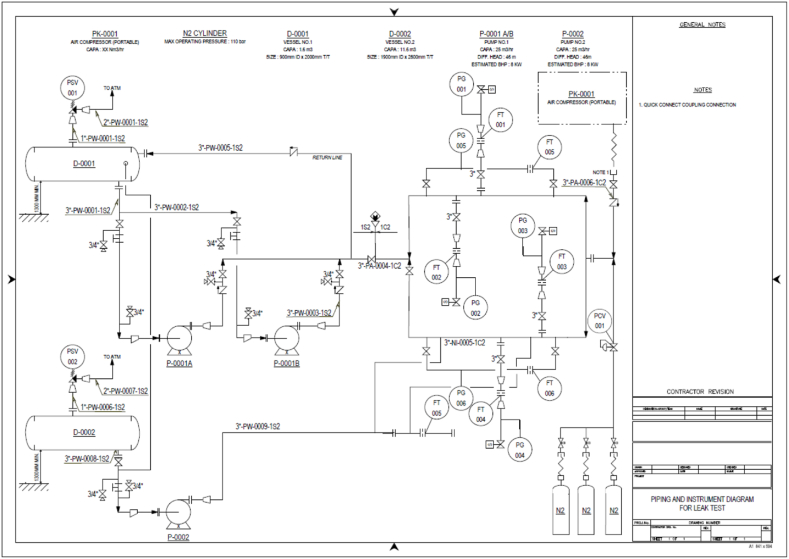
Piping & Instrument Diagram for demonstration experiment site.

**Fig. 8:**
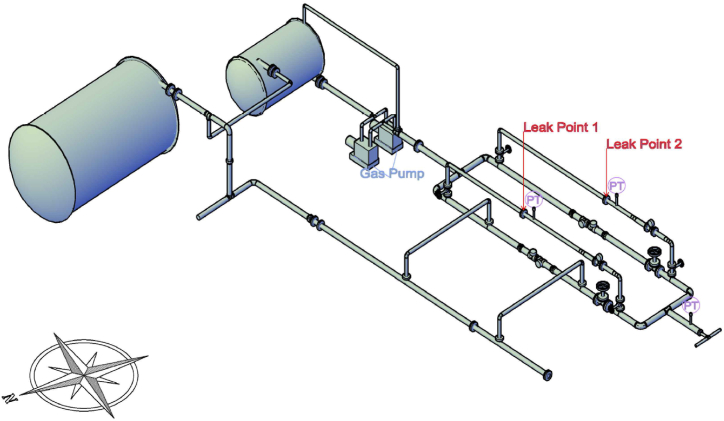
3D Drawing of demonstration experiment site.

The Yeosu and Daesan petrochemical industrial complexes, chosen as representative petrochemical complexes in Korea, were visited in person, and the demonstration experiment site was built through on-site inspection and drawing review. Gas-use processes such as LPG in petrochemical industrial complexes have been applied to the greatest extent possible. The piping was composed of KS D 3562 (carbon steel pipe for pressure piping, SPPS), which is primarily used in the LPG process. Both welded and flanged joints were used for the pipe joint construction. The piping supports were installed with specifications and materials similar to those used in petrochemical plants. Most of the valves in the demonstration experiment site were gate valves, which are commonly used in petrochemical plants. Bent pipes are used in petrochemical plants to mitigate damage due to stress caused by thermal expansion and contraction [[78], [79], [80], [81]]. Various bent shapes such as elbows were incorporated into the piping for the demonstration experiments. Consequently, as shown in Fig. 7, the experiment site was first designed in the form of a Piping & Instrument Diagram (P&ID) for use in petrochemical plants. Based on this, a drawing that applies a demonstration experimental scenario was designed, as shown in the 3D drawing in Fig. 8.

**Fig. 9:**
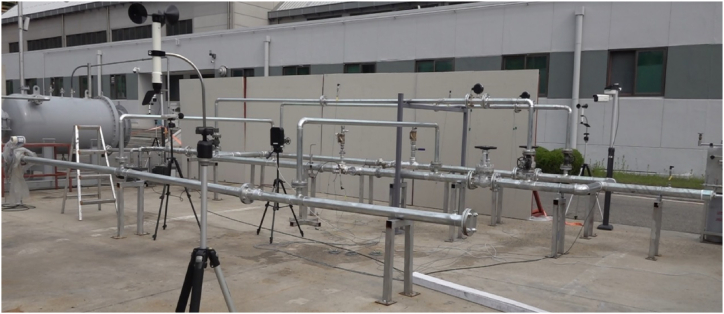
Appearance of demonstration experiment site.

As described in the previous section, an object-detection algorithm for pipe joints was applied to the experimental cases to demonstrate its real-world applicability. As shown in Fig. 9, an outdoor demonstration experiment site was constructed at the Fire Insurer Laboratories in Korea (FILK).

### 6.2 Experiment scenario

An demonstration experiment scenario was developed by identifying the factors that could be sources of noise in petrochemical plants. The noises that could potentially obstruct gas leak detection are as follows.

First, gas pump operation creates noise. Gas pumps in petrochemical plants are a significant source of noise and operate continuously during the process. As listed in Table 4, the noise generated by the gas pump installed during the experiment was approximately 85 dB.

**Table 3:** Cases by scenario.

Case	Leak point^a^	Gas Pump	Barricade	Bolt Loosening			Pressure
1	1	Off	No Wall	0.5 Turn			1.7 bar
				1 Full turn			1.6 bar
				2 Full turns			1.5 bar
2	1	On	No Wall	0.5 Turn			1.7 bar
				1 Full turn			1.6 bar
				2 Full turns			1.5 bar
3	1 & 2, Simultaneously	Off	No Wall	Leak point	1	2 Full turns	1.5 bar
					2		
				Leak point	1	2 Full turns	1.5 bar
					2	0.5 Turn	1.7 bar
				Leak point	1	0.5 Turn	1.7 bar
					2	2 Full turns	1.5 bar
4	1 & 2, Simultaneously	On	No Wall	Leak point	1	2 Full turns	1.5 bar
					2		
				Leak point	1	2 Full turns	1.5 bar
					2	0.5 Turn	1.7 bar
				Leak point	1	0.5 Turn	1.7 bar
					2	2 Full turns	1.5 bar
5	1	On	Wall	0.5 Turn			1.7 bar
				1 Full turn			1.6 bar
				2 Full turns			1.5 bar
6	2	Off	Wall	0.5 Turn			2.6 bar
				1 Full turn			2.5 bar
				2 Full turns			2.5 bar

a See Fig. 8, Leak points 1 and 2.

**Table 4:** Results of the experiment.

Case	Leak point^a^	Pump sound	Pressure			Noise	Detection
1	1	Off (0 dB)	1.7 bar			60 dB	O
			1.6 bar			59 dB	O
			1.5 bar			59 dB	O
2	1	On (85 dB)	1.7 bar			60 dB	O
			1.6 bar			59 dB	O
			1.5 bar			59 dB	O
3	1 & 2, Simultaneously	Off (0 dB)	Leak point	1	1.5 bar	59 dB	O
				2	1.5 bar	59 dB	O
			Leak point	1	1.5 bar	59 dB	O
				2	1.7 bar	60 dB	O
			Leak point	1	1.7 bar	60 dB	O
				2	1.5 bar	59 dB	O
4	1 & 2, Simultaneously	On (85 dB)	Leak point	1	1.5 bar	59 dB	O
				2	1.5 bar	59 dB	O
			Leak point	1	1.5 bar	59 dB	O
				2	1.7 bar	60 dB	O
			Leak point	1	1.7 bar	60 dB	O
				2	1.5 bar	59 dB	O
5	1	On (85 dB)	1.7 bar			60 dB	O
			1.6 bar			59 dB	O
			1.5 bar			59 dB	O
6	2	Off (0 dB)	2.6 bar			83 dB	O
			2.5 bar			82 dB	O
			2.5 bar			82 dB	O

a See Fig. 8, Leak points 1 and 2.

Second, if a gas leak occurs, an ultrasonic wave is generated by the reflection from obstacles surrounding the leak area. The environment was configured so that the reflected ultrasound was intentionally deflected by erecting a wall around the gas leak.

ASTM1002-11 (2022) mandates that the experimental gas should be either nitrogen or compressed air. Thus, nitrogen gas, which is suitable for the experimental environment, was selected [82].

**Fig. 10:**
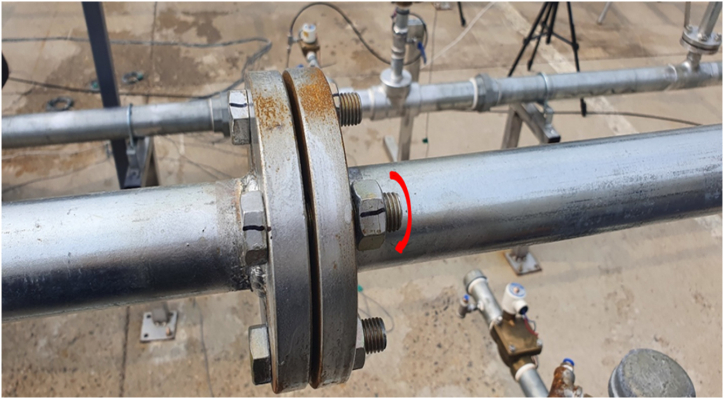
Gas leak point (flange loosened by turning of bolt).

**Fig. 11:**
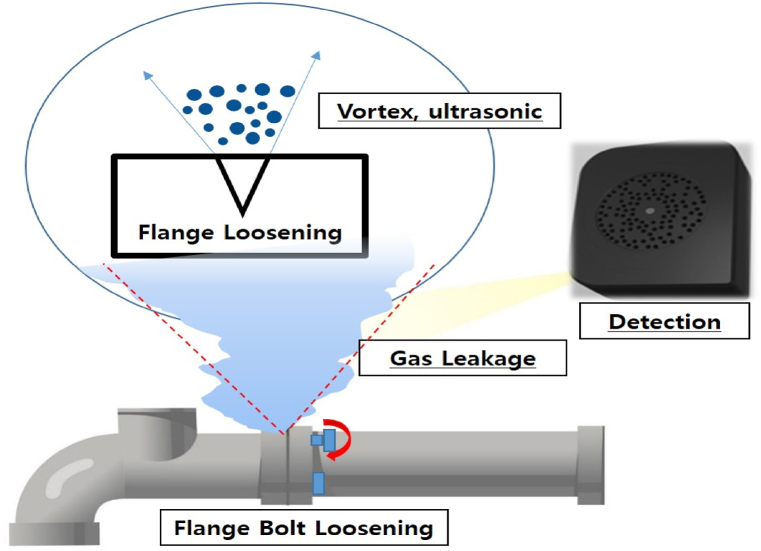
Gas leak Scenario (flange loosening).

As shown in Fig. 10, the selected bolts among the four on the flange of the pipe were carefully loosened by 0.5–2 threads. This bolt loosening detached the flange, replicating a flange leak scenario that can occur in a petrochemical plant, as shown in Fig. 11. The noise levels (dB) generated when leaking from the flange are listed in Table 4 for each case. Simultaneously, obstructions to the ultrasonic detection of gas leaks were created in the surroundings. These obstructions are intentionally generated using various noise sources. The efficacy of the pipe joint detection algorithm was examined in a scenario in which a gas leakage alarm could be falsely triggered by an ultrasonic vision camera because of noise sources.

The camera was strategically placed to capture both the gas leaks and ambient noise in one frame. It is situated to the northwest, more than 2 m from leak point No. 1 and more than 3 m from leak point No. 2, as shown in Fig. 8. To be influenced by the surrounding noise sources, each scenario was located within the corresponding distance range.

During gas leakage, the pressure in the pipe maintains a continuous flow at a medium-low pressure level.

Outdoor demonstration experiments were conducted, as shown in Table 3, under average conditions of a temperature between experiments of 25 ± 1 °C, a wind direction of 199.4° (southwesterly), and a wind speed of 0.9 m/s.

## 7 Experiment and verification

### 7.1 Case #1: one flange gas leakage without noise

**Fig. 12:**
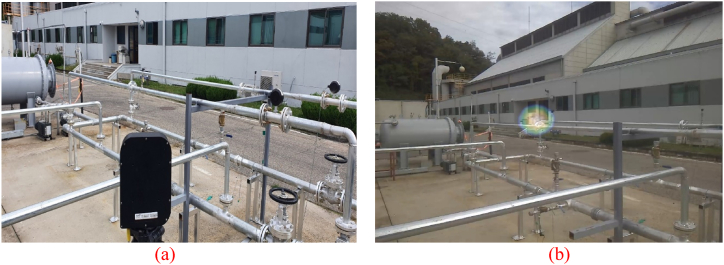
(a) Experiment set-up for case #1. (b) The ultrasonic camera detects the leaky flange without noise.

An ultrasonic camera was positioned to observe the flange as shown in Fig. 12(a). This case was based on an outdoor environment with no running gas pumps or other obstructions.

An intentional gas leak was introduced at the flange by loosening the bolt and maintaining pressure inside the pipe. The result of the leakage from only one point in the piping system without the surrounding pump noise or reflected noise is shown in Fig. 12(b).

In this case, the same results appear when the AI algorithm that removes the surrounding noise is applied and when it is not. However, if surrounding noise occurs, it can be effectively removed by using a pipe-joint object-detection algorithm. It is possible to check where the AI algorithm is applied in case #2, which includes gas pump noise in the scenario.

### 7.2 Case #2: one flange gas leakage with direct noise from the gas pump motor

**Fig. 13:**
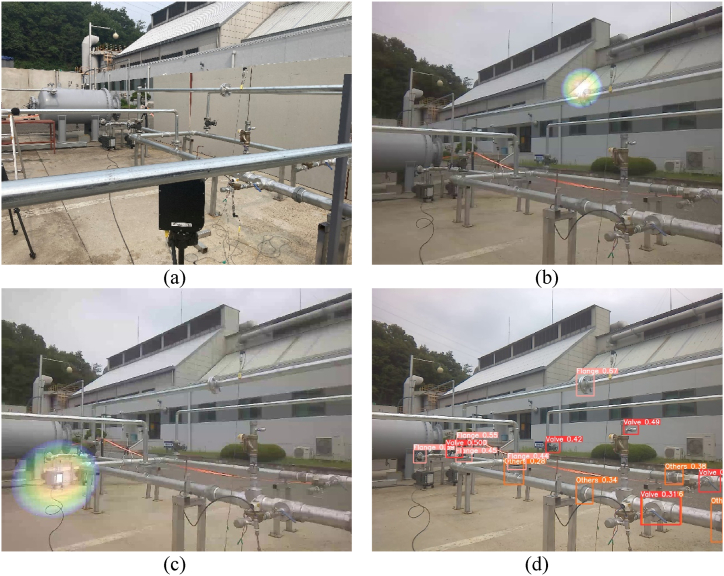
(a) Experiment set-up for case #2. (b) The ultrasonic camera detects the leaky flange without noise. (c) The ultrasonic camera output shows that the gas pump works as a noise source. (d) Pipe joint detection result from the image taken by the ultrasonic camera.

An ultrasonic camera was positioned to observe the two gas pumps and the flange, as shown in Fig. 13(a). An intentional gas leak was introduced at the flange by loosening the bolt and maintaining pressure inside the pipe. Under normal circumstances, an ultrasonic camera can easily detect leaks, as shown in Fig. 13(b).

One of the gas pumps was turned on, and the motor inside the gas pump generated noise. The ultrasonic camera identified the gas pump as a major sound source, as shown in Fig. 13(c), by overlapping a heat map over the working gas pump, but not over the leaky flange. This can trigger a false alarm for safety managers, which requires unnecessary time and effort and eventually degrades confidence in the leakage monitoring system. Furthermore, the actual leakage cannot be detected or properly reported.

A pipe joint object-detection algorithm was applied to the visual images obtained using the ultrasonic camera (Fig. 13(d)). The detection algorithm detects and draws bounding boxes across the three types of joints (flange, valve, etc.) observed in the image based on their confidence scores. This detection is sufficient to identify the flange at which the gas leaks, whereas gas pumps are not regarded as pipe joints. It was possible to eliminate the sound signals originating from the direction of the gas pump. If there is an actual leak, as in this case, it can be detected despite the gas-pump noise, and if there is no leak, false alarms can be prevented.

### 7.3 Case #3: two flange gas leakages without noise

**Fig. 14:**
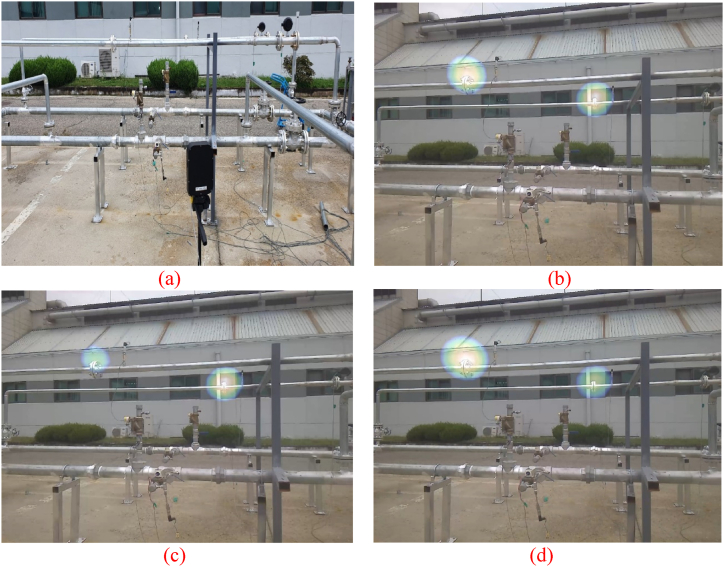
(a) Experiment set-up for case #3; (b) 1.5 bar each at the left leak point (leak point 1 in Fig. 8) and right leak point (leak point 2 in Fig. 8); (c) 1.5 bar at the left leak point, and 1.7 bar at the right leak point; (d) 1.7 bar at the left leak point, and 1.5 bar at the right leak point.

An ultrasonic camera was used to observe the two flanges as shown in Fig. 14(a). An intentional gas leak was introduced at the two flanges by loosening the bolt and maintaining the pressure inside the pipe. The gas leaks were generated under the same pressure conditions (Fig. 14(b)) and different pressures. The different pressure conditions include cases in which the pressure on the left flange is lower (Fig. 14(c)) or higher (Fig. 14(d)) than that on the right flange.

To the best of our knowledge, there are very few cases in which leaks occur simultaneously in two or more parts, other than those caused intentionally. However, the possibility of a leak occurring in another part cannot be ruled out when performing work involving a gas leak. Therefore, it is meaningful to generate two leaks simultaneously and identify whether the developed system can detect simultaneous leaks.

In case #3, there was no nearby noise source such as the pump noise in case #2. Therefore, in case #2, the pipe joint object detection algorithm could be applied effectively; however, in case #3, the performance of the ultrasonic camera was more important than that of the AI algorithm.

Gas leaks occurring at two locations simultaneously were well detected not only at the same pressure and noise level (Fig. 14(b)), but also at different pressure and noise levels (Fig. 14(c) and (d)). Although there were some differences in the expression of the gas leak depending on the pressure and resulting noise level, it was displayed on the heat map at a level that could be perceived well by humans.

### 7.4 Case #4: two flange gas leakages with noise from the gas pump motor

**Fig. 15:**
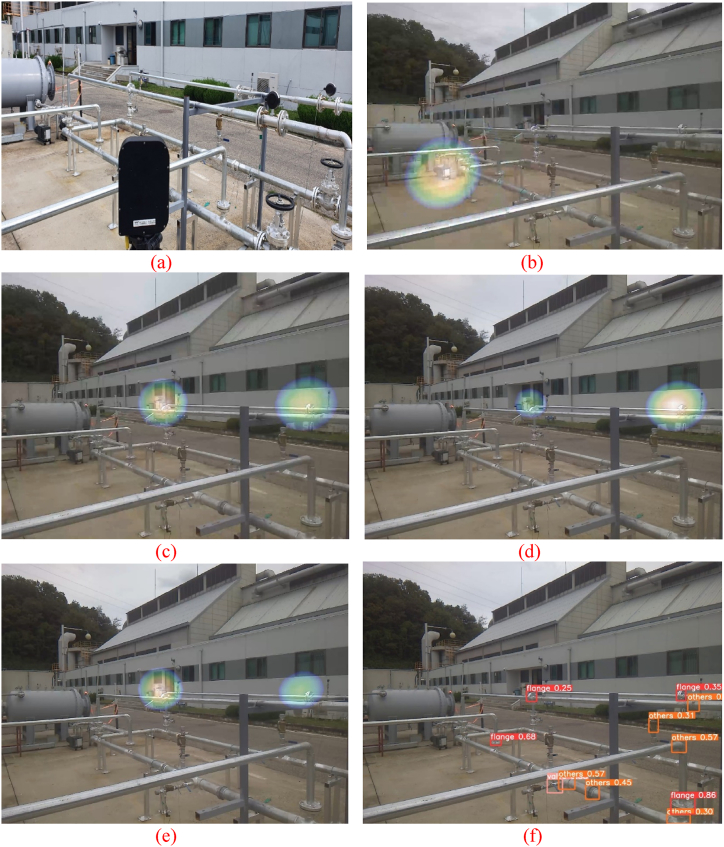
(a) Experiment set-up for case #4. (b) The ultrasonic camera output showing that the gas pump works as a noise source. (c) 1.5 bar each at the left (leak point 1 in Fig. 8) and right (leak point 2 in Fig. 8) leak points. (d) 1.5 bar at the left leak point, and 1.7 bar at the right leak point. (e) 1.7 bar at the left leak point, and 1.5 bar at the right leak point. (f) Pipe joint detection result from the image taken by the ultrasonic camera.

An ultrasonic camera was positioned to observe the gas pump and the two flanges, as shown in Fig. 15(a). An intentional gas leak was introduced at the flange by loosening the bolt and maintaining pressure inside the pipe. Under normal circumstances, an ultrasonic camera can detect leaks, as shown in Fig. 15(c) and (e).

One of the gas pumps was turned on, and the motor inside the gas pump generated noise. The ultrasonic camera identified the gas pump as the major sound source, as shown in Fig. 15(b), by overlapping a heat map over the working gas pump, but not over the leaky flange.

As shown in Fig. 15(f)–a pipe joint object detection algorithm was applied to the visual images obtained from the ultrasonic camera. The detection algorithm detects and draws bounding boxes across the three types of joints (flange, valve, etc.) observed in the image based on their confidence scores. This detection is sufficient to identify the flange at which the gas leaks, whereas gas pumps are not regarded as pipe joints. It was possible to eliminate the sound signals originating from the direction of the gas pump. Similar to case #2 and case #4, the detection of an actual leak was possible despite the gas pump noise. Detection was considered successful when two actual leakages occurred simultaneously.

An additional experiment was conducted under the same conditions under which the two leaks occurred at different times. After creating one leak, another occurred at a different point. This additional experiment also confirmed that both leaks were successfully detected even though they occurred at different times.

### 7.5 Case #5: Indirect noise from the gas pump motor

**Fig. 16:**
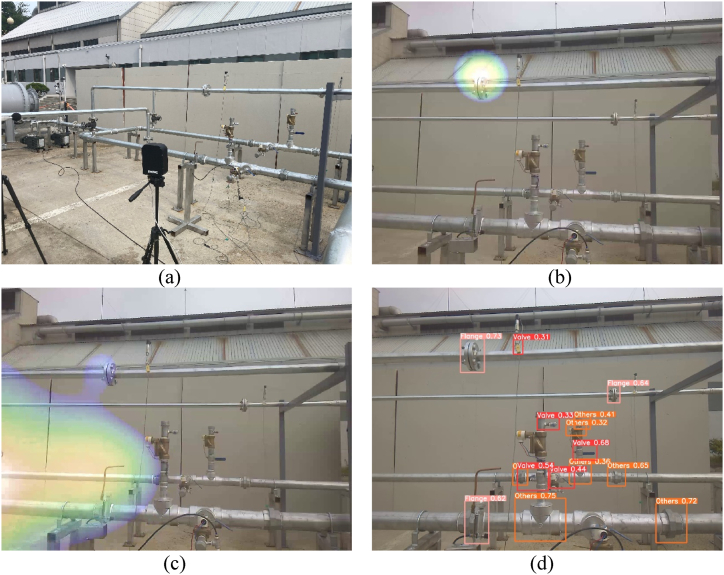
(a) Experiment set-up for case #5. (b) The ultrasonic camera detects the leaky flange without noise. (c) The ultrasonic camera output showing that the sound reflected by the safety wall works as a noise source. (d) Pipe joint detection result from the image taken by the ultrasonic camera.

An ultrasonic camera was used to observe the flange and the safety wall, as shown in Fig. 16(a). The gas pumps were placed outside of the view of the ultrasonic camera. An intentional gas leak was introduced at the flange by loosening the bolt and maintaining pressure inside the pipe. Under normal conditions, the ultrasonic camera could easily detect leakage, as shown in Fig. 16(b).

In case #1, one of the gas pumps was turned on, but the motor sound was reflected by the safety wall, which acted as a noise source. The ultrasonic camera identified the reflected sound originating from the wall, as shown in Fig. 16(c). The sound induced by the actual leak was also weakly detected and drawn in blue over the flange; however, the actual leak was easily missed.

A pipe joint object-detection algorithm was applied to the visual images obtained using the ultrasonic camera (Fig. 16(d)). The detection algorithm accurately detects the joints within an image. When noise signals from an area without pipe joints are suppressed, false alarms can be prevented, and actual leaks can be detected.

### 7.6 Case #6: reflected sound from an actual gas leakage

An ultrasonic camera was positioned to observe the flange and safety wall, as in case #2. We introduced an intentional gas leak into the system by repeating the procedure described for case # 1. At slightly higher pressures than those in cases #1 and #2, a noise source was formed due to reflection. Under normal conditions, the ultrasonic camera could easily detect leakage, as shown in Fig. 12, Fig. 13 and 16(b).

**Fig. 17:**
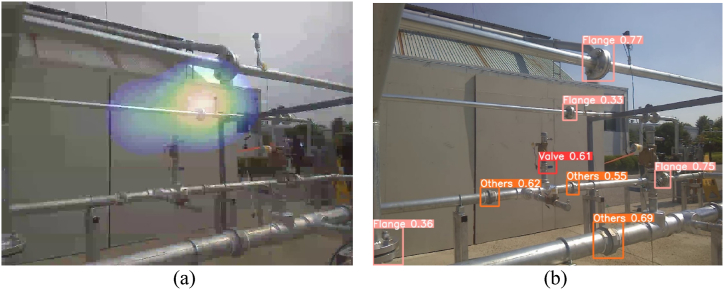
(a) The ultrasonic camera detects both the actual leakage and its reflection, as if there are two leaks. (b) Pipe joint detection result from the image taken by the ultrasonic camera.

In this case, the ultrasonic sound generated by the leak is reflected by the safety wall and becomes a source of noise. The ultrasonic camera identified the actual leak and reflected sound, which may be seen as two nearby leaks (Fig. 17(a)).

A pipe joint object detection algorithm was applied to the visual images obtained from the ultrasonic camera (Fig. 17(b)). When the noise signals from the area without pipe joints were suppressed, only the actual leak remained, thereby eliminating further confusion.

## 8 Discussion

We anticipate that, using the results of this study, we will be able to prevent accidents and mitigate risks by detecting gas leaks in pipes within petrochemical industrial complexes. Unlike current commercialized portable models, the proposed system is structured and nonportable. Moreover, it allows for the constant monitoring of gas leak risk areas in real time. If this system is implemented within a petrochemical industrial complex, we expect that damage can be minimized more effectively through prompt leak detection and response.

The integration of AI with ultrasonic detection technology provides a significantly more accurate and faster method for detecting gas leaks. The pipe joint object detection algorithm can be effectively applied not only to the ambient noise set in the demonstration experiment, but also to unexpected noises caused by transportation, people, air currents, and aircraft movement. This innovation can improve the process safety by identifying potential hazards before they become major incidents. AI can also be applied to identify anomalies in real-time to support preventive maintenance and operational efficiency. The integration of AI can increase the chances of detecting gas leaks and is always better than current petrochemical plants that do not have an ultrasonic-based pipe leakage monitoring system. However, possible misses in detection can sometimes be critical in real situations; thus, efforts to improve the performance of AI models must be made for better safety. To further reduce misses in object detection, more industrial pipe images are being collected, and more pipe joint objects are being labeled. Existing labels are also being refined. Based on the characteristics of ultrasonic signals owing to pipe leakage, finding the local maxima of the ultrasound intensity data over a field of view that lasts for a few seconds can be a good heuristic method for locating possible leakage points.

However, possible misses in detection can sometimes be critical in real situations; thus, efforts to improve the performance of AI models must be made for better safety. To further reduce misses in object detection, more industrial pipe images are being collected and more pipe joint objects are being labeled. Existing labels are also being refined. Furthermore, it is possible to set up a second line of defense because the proposed system finds the local maxima over the entire field of view. To ensure the safety of petrochemical plants, it is necessary to develop a fail-safe system with protection from multiple layers. One possible additional method is to exploit the characteristics of ultrasonic signals owing to pipe leakage. Finding the local maxima of ultrasound intensity data that consistently last for a few seconds can be a good heuristic method for locating possible leakage points. This is not possible if object detection is performed first, and the ultrasound intensity data do not exist at other locations over the field of view, as in a previous study [73].

This technology can be designed to work in conjunction with existing detection systems to provide an additional line of defense. Interoperability also enables cross-validation, which improves the reliability of the leak detection. The concepts of crosschecking and failsafe safety management can be applied in conjunction with existing gas leak detection systems.

In addition, if this technology is combined with an interlock system, which is a petrochemical plant control system, additional damage can be prevented by isolating the leaked area immediately upon leak detection.

This technology, which combines AI with petrochemical plants, can collect and eliminate misrecognized events by applying plant site characteristics. Thus, it is possible to develop a suitable system for each site.

Using this technology, petrochemical plants can potentially reduce their downtime. It can protect the health of employees and nearby residents and prevent environmental damage associated with gas leaks.

In addition, continuous surveillance can provide valuable data for risk assessment, the implementation of proactive safety measures, and the improvement of emergency response plans.

Similar to existing CCTV systems, the proposed system has the potential to be implemented in various workplaces. This is expected to increase safety within the petrochemical industrial complex and extend to various other industries, thereby reducing damage by applying developed products across different sectors.

The research and development undertaken is significant because it represents the first instance of combining ultrasonic detection technology, which is capable of detecting microleaks, with image-object detection using AI technology. In addition, because a large amount of data can be collected, it can be used for further research.

## 9 Conclusion

In this study, we developed a stationary gas-leak monitoring system that integrates ultrasound and AI technologies. An AI algorithm was employed to enhance the precision of pipe feature detection, thereby decreasing the false recognition rate of ultrasonic detection. A demonstration experiment was conducted using an AI trained in an ultrasonic sensing system.

(1) Following training using YOLOv5 based on web crawling and photos taken directly, our AI achieved an mAP50 score of 0.45 for all classes. We used a pipe-detection algorithm to identify the joints and points of failure. Therefore, low mAP50 scores are not a major concern as the majority of potential joints within an image can be detected, which is the case with our application.
(2) Site visits and reviews were conducted at several petrochemical plants. Based on a preliminary investigation, an outdoor demonstration site similar to a petrochemical plant was designed and manufactured. The site includes vessels, gas pumps, piping systems, instrumentation, and obstructions.
(3) An experimental scenario was prepared using the literature and case studies. This includes the source and location of the gas leak as well as surrounding environmental noise factors.
(4) In the demonstration experiment, scenarios involving ambient noise sources that can occur in petrochemical plants were generated. Despite these obstacles, we verified that the actual leakage source could be identified.
(5) A demonstration experiment was conducted using this R&D system, which applied trained AI to an ultrasonic camera. These studies confirmed that the ambient noise source could be removed, and the actual gas leak could be detected using the AI piping detection technique.

Further research is required to address the challenges associated with higher AI precision and harsher ambient noise sources through additional learning and demonstration experiments.

## Additional information

No additional information is available for this paper.

## Data availability statement

No additional information is available for this study. However, data will provided upon reasonable request.

## Declaration of ethics

Informed consent was not required for this study because it focused only on technical system development issues. Not applicable.

## CRediT authorship contribution statement

**JoonHyuk Lee:** Writing – review & editing, Writing – original draft, Validation, Methodology, Funding acquisition, Formal analysis, Conceptualization. **YoungSik Kim:** Writing – original draft, Visualization, Software, Data curation, Conceptualization. **Abdur Rehman:** Visualization, Software, Data curation. **InKwon Kim:** Visualization, Resources, Investigation, Formal analysis. **JaeJoon Lee:** Writing – review & editing, Supervision, Methodology, Funding acquisition, Conceptualization. **HongSik Yun:** Writing – review & editing, Supervision, Resources, Project administration, Conceptualization.

## Declaration of competing interest

The authors declare that they have no known competing financial interests or personal relationships that could have appeared to influence the work reported in this paper.
